# HIV/Hepatitis C Virus Co-infection among Adults Beginning Antiretroviral Therapy, Malawi

**DOI:** 10.3201/eid2211.160892

**Published:** 2016-11

**Authors:** Münevver Demir, Sam Phiri, Rolf Kaiser, Thom Chaweza, Florian Neuhann, Hannock Tweya, Gerd Fätkenheuer, Hans-Michael Steffen

**Affiliations:** University Hospital of Cologne, Cologne, Germany (M. Demir, R. Kaiser, G. Fätkenheuer, H.-M. Steffen);; Lighthouse Clinic, Lilongwe, Malawi (S. Phiri, T. Chaweza, H. Tweya);; Institute of Public Health, University Hospital of Heidelberg, Heidelberg, Germany (F. Neuhann) ;; German Centre for Infection Research, Cologne, (G. Fätkenheuer)

**Keywords:** HIV/HCV co-infection, prevalence, Malawi, viruses, HIV/AIDS and other retroviruses, hepatitis C

**To the Editor:** Throughout the world, ≈115 million persons have hepatitis C virus (HCV) antibodies, ≈37 million are infected with HIV type 1, and an estimated 2.3 million persons are infected with both viruses (*1*). The estimated prevalence of HIV infection among adults in Malawi is 9.1% (*2*). Data concerning HCV seroprevalence in Malawi are conflicting and range from 0.0% to 18.0%, depending on the studied population and the chosen methods for HCV infection diagnosis (*3*–*6*). In a recent study, researchers used stored blood samples (without HCV confirmatory assays) from studies in rural and urban Malawian populations (1989–2008); an HCV seroprevalence of 6.8% was found in HIV-positive patients (*7*). In contrast, in a cohort of HIV-negative mothers (2006–2010), only 0.5% were found to be HCV positive with confirmatory HCV testing by immunoblot (*8*). These studies were not included in a 2015 metaanalysis that estimated the seroprevalence of HCV infection and HIV/HCV co-infection in Malawi to be 7.7% and 2.0%, respectively (*9*). Liver disease progresses more rapidly in HIV/HCV co-infected patients than in HCV monoinfected patients (*10*), and the highly effective second-generation direct-acting antiviral therapies are less toxic than interferon-based treatment regimens. It is crucial to gather accurate epidemiologic information on the burden of HIV/HCV co-infection to support the design and implementation of HCV treatment initiatives in resource-limited settings such as sub-Saharan Africa.

The aim of our study was to evaluate the prevalence of HIV/HCV co-infection in HIV-positive adults. We used baseline data obtained during the first 4 months of the ongoing observational, prospective Lighthouse Tenofovir Cohort (LighTen) study (Clinicaltrials.gov identifier: NCT02381275), which is conducted at the Lighthouse Clinic in Lilongwe, Malawi, in cooperation with the Institute of Public Health, University of Heidelberg, Germany, together with the Division of Infectious Diseases and the Clinic for Gastroenterology and Hepatology, University Hospital of Cologne, Germany. Ethical approvals by the respective committees of the Research Commission of the Ministry of Health, Malawi, and the participating German universities were granted before the study was initiated. A patient was included if he or she had confirmed HIV infection, was >18 years of age, and had given written informed consent for study participation. We included patient demographic information, medical history, concomitant diseases, bodyweight and height, as well hepatic panel results, platelet count, estimated glomerular filtration rate (determined by the CKD Epi formula), hemoglobin level, leukocyte count, CD4 cell count, and quantitative HIV RNA (Roche COBAS TaqMan HIV-1 v2.0, Risch, Switzerland) recorded at patient enrollment from the Kamuzu Central Hospital, Lilongwe, Malawi.

Aliquots of blood samples were stored at −80°C and kept on dry ice during airfreight to Cologne, then stored at −80°C until testing. Samples were thawed and tested for HCV IgG by a chemiluminescent micro-particle immunoassay (Abbott ARCHITECT, Wiesbaden, Germany) at the Institute of Virology, University of Cologne, Germany. Specimens that reacted to this immunoassay underwent supplemental testing with PCR for HCV RNA by a quantitative assay (Abbott RealTime HCV). We analyzed baseline characteristics of the study population (Table) using descriptive statistics (SPSS software version 22; IBM Inc., Chicago, IL, USA).

**Table:** Characteristics of HIV-positive adults with HIV/HCV co-infection, Malawi*

Characteristic	HIV-positive and HCV antibody–negative patients, n = 222	HIV-positive and HCV-antibody–positive patients, n = 5
Age, y	36.1 (30.5–41.5)	34.5 (24.8–50.2)
Sex, M/F	88/134 (40/60)	2/3 (40/60)
Weight, kg, n = 167	59.1 (52.7–66.7)	59.6 (55.9–68.7)
Height, cm, n = 170	157.0 (151.0–165.0)	159.5 (151.0–167.0)
Quantitative HIV RNA [<40] × 10^3^, copies/mL, n = 205	44.43 (12.09–158.96)	30.87 (21.14–95.38)
History of blood transfusion	21 (9)	1(20)
History of jaundice	2 (1)	0
eGFR mL/min/1,73 qm, n = 194	96.0 (79.0–110.0)	91.0 (88.0–94.0)
ALT [7–35], IU/L, n = 183	23.3 (14.9–33.4)	15.6 (13.2–22.5)
AST [≤38], IU/L, n = 183	31.7 (23.0–42.4)	31.9 (23.2–43.7)
Total bilirubin level [≤1.3], mg/dL, n = 185	0.30 (0.20–0.45)	0.26 (0.20–0.48)
Platelet count [122–330] × 10^9^/L, n = 214	226 (181–293)	283 (160–320)
Hemoglobin level [10.9–17.3], g/dL, n = 215	12.4 (10.8–13.7)	13.3 (11.8–15.1)
Leukocyte count [2,800–8,400], cells/µL, n = 213	4,400 (3,500–5,400)	3,900 (2,700–6,600)
CD4 cell count, cells/µL, n = 139	284 (101–421)	319 (189–449)

*Values are given as total no. (%) or as median with interquartile range for the HCV antibody–negative study population and median with range for the HCV antibody–positive study population, respectively. Reference values are given in brackets. Number of missing values for the HCV-antibody negative patients ranged from 88 to 3, indicated by differing patient numbers in stub column. ALT, alaninine aminotransferase; AST, aspartate aminotransferase; eGFR, estimated glomerular filtration rate; HCV, hepatitis C virus.

All 227 patients (137 female, median age 36.1 years) were HIV positive, with a median quantitative HIV RNA of 44,389 copies/mL and median CD4 cell counts of 284 cells/µL. Twenty-two patients (9.7%) had a history of blood transfusion, and 0.9% had a history of jaundice. Results for alanine aminotransferase level, aspartate aminotransferase level, total bilirubin level, and platelet counts were within the reference range in almost all patients, and no patient was jaundiced. Five patients (2.2%) had HCV IgG. However, none of these patients had detectable HCV RNA. Thus, the prevalence of active HIV/HCV co-infection was 0% in the studied cohort. One of the 5 patients who were positive for IgG against HCV also had a history of blood transfusion; none had a history of jaundice, and all but 1 seropositive patient had liver function tests within the reference range.

Three studies in Malawi have used PCR to test for HCV: 2 studies in HIV-positive pregnant women (included in the aforementioned metaanalysis) (*9*) and 1 study in blood donors (*5*), of whom 10.7% were HIV infected. PCR results for HCV RNA were positive in 0/2,041 (0%), 1/309 (0.3%), and 1/140 (0.7%) of these cases. Our findings confirm this low prevalence of PCR-positive active HCV infection also among HIV-infected adults from the general population of urban Lilongwe. Together, these studies indicate an overestimation of HCV prevalence on the basis of screening assays (*7*). 

From a public health point of view, HCV infection as a cause of liver-related illness seems of minor importance in Malawi. The fact that HCV prevalence in antenatal care cohorts with already established serum sampling for routine HIV testing is similar to that in this mixed cohort of urban Malawi residents suggests that samples from these programs might represent a cost-efficient opportunity for monitoring trends of HCV infection in the population.
